# Lobular Capillary Hemangioma in a Young Child: Diagnostic Dilemma and Management

**DOI:** 10.7759/cureus.44966

**Published:** 2023-09-09

**Authors:** Cheranjeevi Jayam, Immaculate Jonna, Navaneeth Yerragudi, Jitendra Chawla

**Affiliations:** 1 Dentistry, All India Institute of Medical Sciences, Mangalagiri, IND

**Keywords:** mandibular gingival region, case report, excisional biopsy, very young child, large pyogenic granuloma

## Abstract

Pyogenic granuloma (lobular capillary hemangioma) is a common reactive tumour-like lesion of the oral cavity arising in response to various stimuli such as chronic local irritation, drug intake, and hormonal changes. The clinical features are similar to other reactive oral lesions such as peripheral giant cell granuloma (PGCG) and therefore the definitive diagnosis depends on histopathologic evaluation. We report a case of a three-year-old Indian boy presenting with a three-month history of a 3.3×1.4×0.8 cm large sessile, lobular, soft-tissue mass of the left mandibular posterior gingival region which was referred to us. An excisional biopsy of the lesion revealed multinucleated giant cells lying in an inflammatory cell infiltrate-rich stroma consisting of plump endothelial cells on histopathologic examination suggestive of lobular capillary hemangioma. The patient was asymptomatic with no new growth on regular follow-up. This is the first reported case of a large pyogenic granuloma in a very young child, which is an uncommon presentation.

## Introduction

The pyogenic granuloma (PG) or lobular capillary hemangioma (LCH), first described by Hartzell, is a non-neoplastic tumour-like growth of the oral cavity [1]. It is most commonly found in the gingiva followed by other areas such as lips, tongue, and buccal mucosa [2]. In children, reactive oral lesions like PG can proliferate and become symptomatic; therefore, prompt diagnosis and treatment are of utmost importance. LCH can be often mistaken for commonly occurring periapical pathologies or vestibular abscesses especially when present adjacent to carious teeth. Certain other soft-tissue growths that mimic PG in this age group are peripheral giant cell granuloma (PGCG), peripheral ossifying fibroma, and fibrous epulis [3]. Negative behaviour in very young children is an impediment to proper clinical examination leading to wrong diagnosis sometimes. This case report deals with a large lesion in a very young child where the primary objective was the resolution of the lesion without any symptoms after correct diagnosis and treatment.

## Case presentation

**Figure 1:**
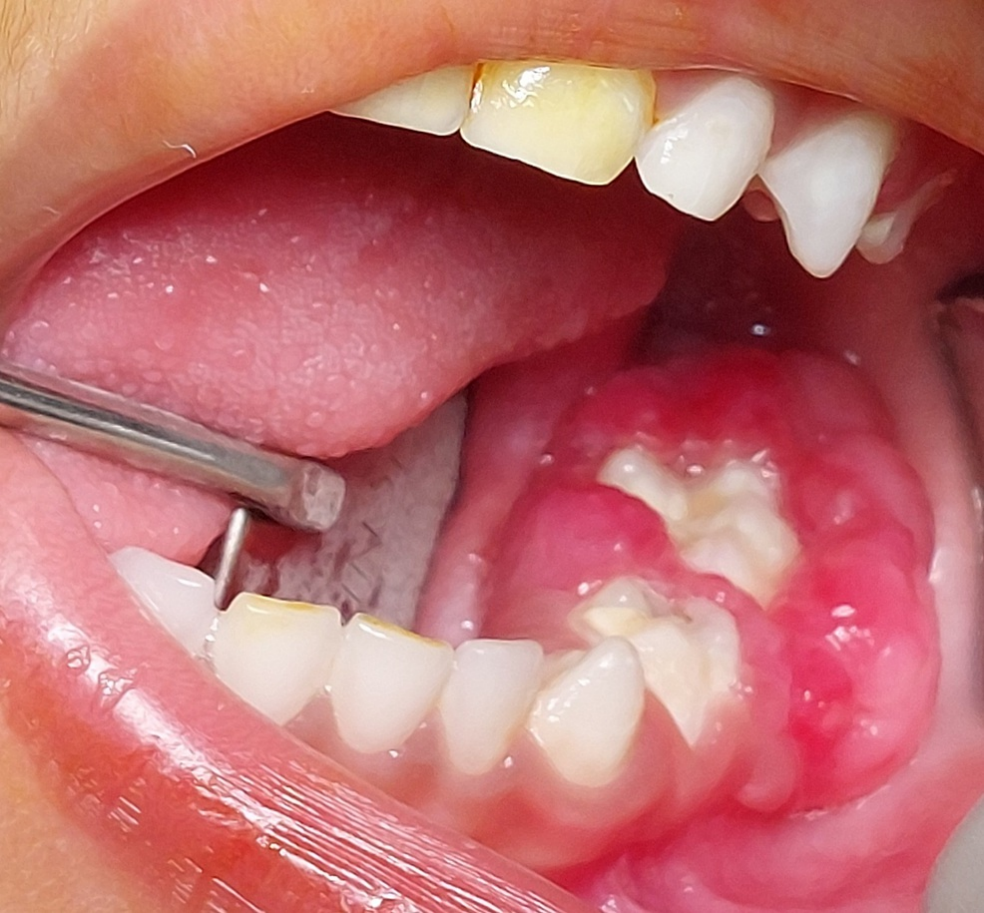
Clinical image of the lesion

A three-year-old Indian boy reported to our department with a large gingival swelling and difficulty in mastication. The child was prescribed antibiotic courses several times at multiple clinics. Non-resolution of the lesion compelled parents to seek treatment at our hospital. Parents also reported that the child’s negative behaviour hampered proper intraoral examination and hence proper diagnosis. Clinical evaluation of the patient in our hospital was done in a child-friendly environment; intraoral examination revealed the presence of a reddish pink sessile, lobular mass of size 3.3×1.4×0.8 cm in the left posterior mandibular region which was soft to firm on palpation (Figure 1).

The lesion involved the gingiva around the mandibular first and second primary molar and obliterated the buccal and lingual vestibule. It had started as a small lobe which gradually increased in size to reach the present size and the patient had difficulty in food consumption as reported by the parent. There was dental plaque and the presence of caries in teeth adjacent to the lesion was noted as well. A differential diagnosis of PG or PGCG based on clinical presentation was made.

Laboratory tests and results

**Figure 2:**
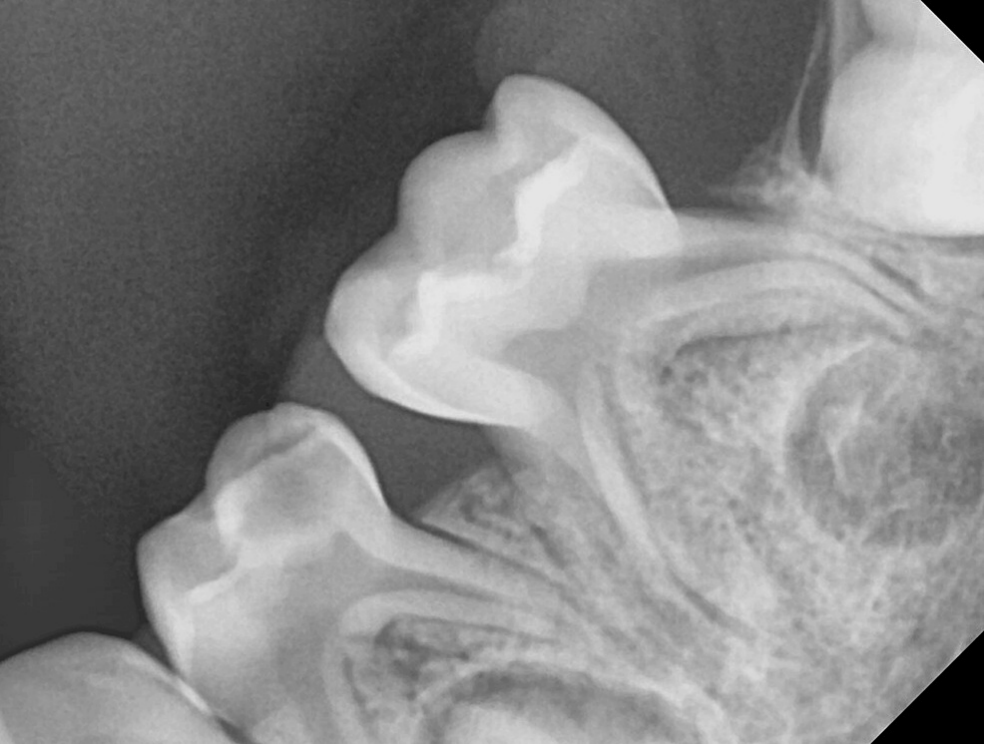
Radiographic image of the involved area

There was superficial erosion of the alveolar bony crest in relation to the molar teeth. The second primary molar showed a circumscribed radiolucent lesion surrounding the distal root apex despite being caries-free. The striking feature of PGCG is the superficial resorption of the bony crest or cupping defect [4]. The first molar showed a deep carious dentinal lesion as well (Figure 2).

Pain-eliciting pulp sensibility test to ascertain pulp involvement at the clinic was not possible due to young age. Hence, the presumptive diagnosis of PGCG was made.

Treatment and follow-up

**Figure 3:**
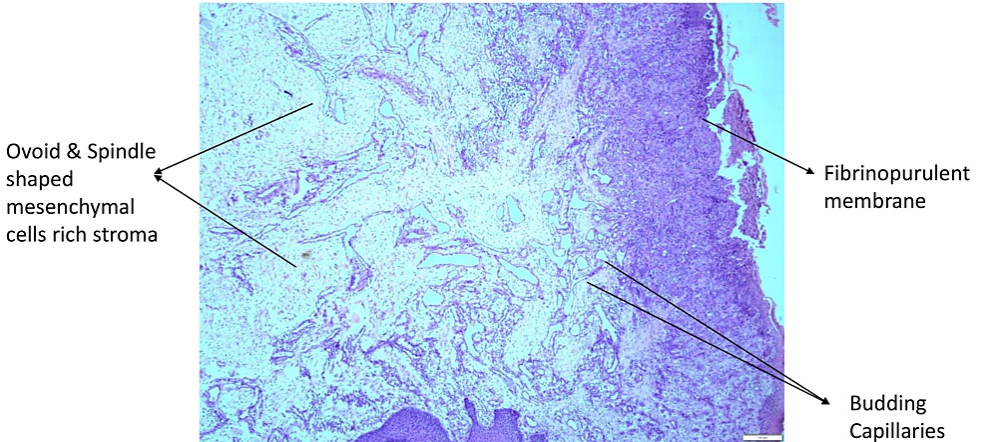
Histopathological image 1

**Figure 4:**
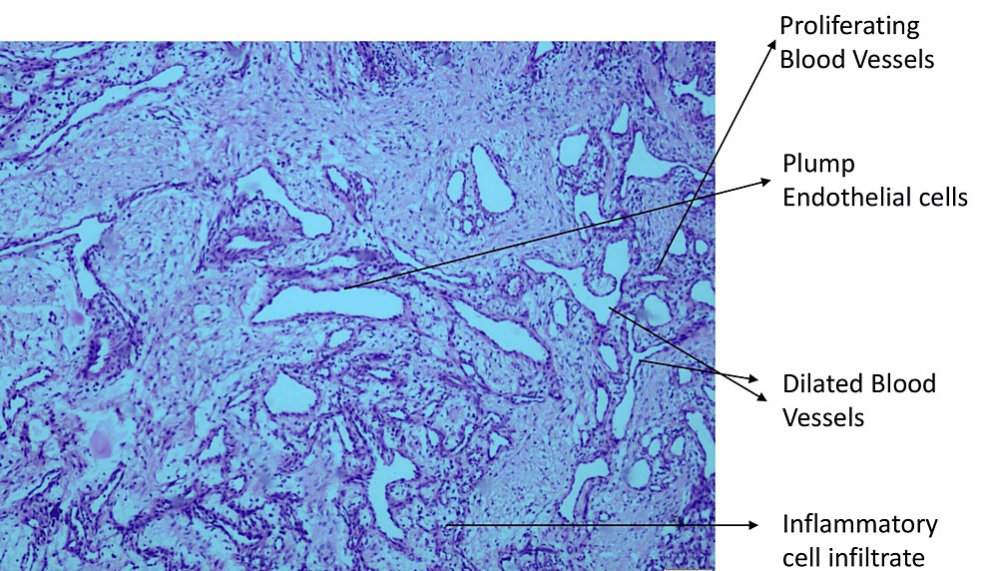
Histopathological image 2

Treatment was initiated after obtaining informed consent from the parents after explaining the condition, side effects, and benefits and risks of treatment. In the initial appointment, restorations were done using the atraumatic restorative technique to improve the otherwise negative behaviour of the child at 2-3 years of age. Oral prophylaxis was done and the area of the lesion was cleaned and irrigated to decrease the inflammation around the lesion. Multiple irrigations were done locally every three days for two weeks to decrease inflammation and improve oral hygiene before attempting treatment. The carious teeth were restored. Later, the lesion was surgically excised under general anaesthesia after evaluating the patient’s general condition and suitability to undergo the treatment. Excisional biopsy was performed and histopathological diagnosis revealed acanthotic epithelium, several dilated, abundant proliferative blood vessels lined by flattened-to-plump endothelial cells, and an inflammatory cell infiltrate-rich stroma, which was suggestive of LCH (Figures 3, 4).

**Figure 5:**
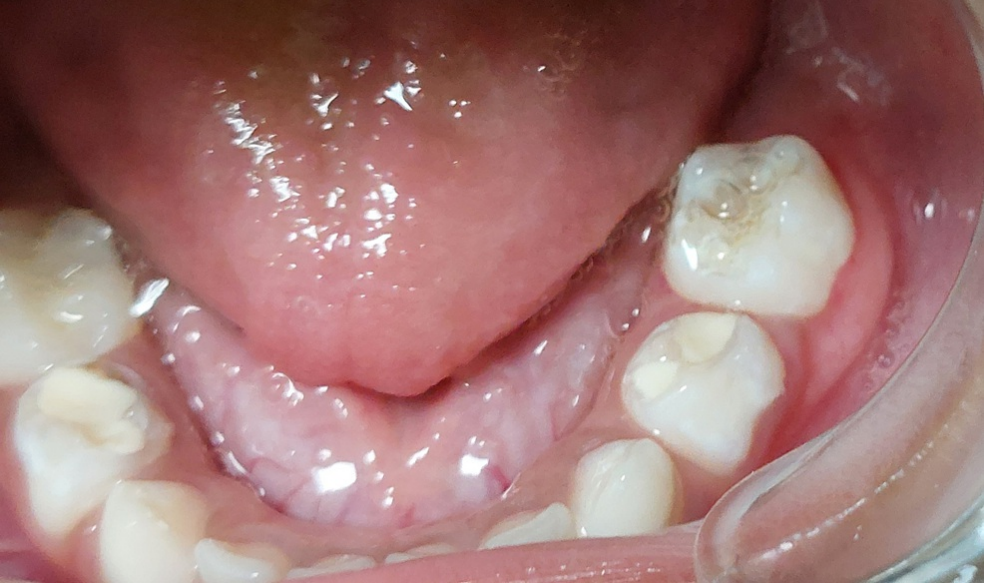
Image depicting the complete resolution of the lesion

In the one-month postoperative follow-up visit, the area showed complete resolution of the lesion (Figure 5).

Regular follow-up visits were done at seven days, 15 days, one month, and three months, respectively. The patient will be recalled at six months and every year hereafter. The importance of maintaining good oral hygiene was also emphasised to the parents.

## Discussion

PG occurs as a long-term response to factors such as localised irritation, trauma, and hormones. Its occurrence is rather uncommon in the oral cavity of children, with poor oral hygiene being the common causative factor [5,6]. Peak incidence is in the second to fifth decade of life [7].

Differential diagnoses for gingival lesions in children that may mimic the PG are the PGCG, peripheral ossifying fibroma, parulis, hemangioma, and fibrous epulis [3]. Based on the clinical features alone, it may be difficult to differentiate PG from other lesions. PG presents as a friable, soft nodule that bleeds easily with manipulation and is non-aggressive whereas the PGCG is more often either reddish pink or bluish-red to purple coloured and might cause bone resorption. The PGCG most commonly occurs on the gingiva and alveolar mucosa [8]. Although the PGCG develops within the soft tissue, it sometimes presents as a "cupping" superficial resorption of the underlying alveolar bony crest [9]. PG normally does not show any radiographic presentations. However, rarely, it might present as localised alveolar bone resorption in long-standing cases [10]. Both PG and PGCG have a similar clinical presentation and long-standing poor oral hygiene could stimulate their occurrence. Hence, there was a diagnostic dilemma prior to histopathological evaluation in our case as well. The definitive difference is the marked presence of numerous giant cells on histopathological evaluation in the case of PGCG [9]. 

The parulis is an erythematous nodule of the gingiva, which is frequently associated with a foreign body, a pocket, or a non-vital tooth. It is generally painful with purulent exudate, which helps to differentiate this inflammatory disease from PG [3]. The peripheral ossifying fibroma is often ulcerated and inflamed but lacks purplish-blue discolouration. It commonly occurs on the interdental papilla and may be pedunculated or broad-based. The colour may vary from pale pink to cherry red. It is also considered to be a mature version of PG and PGCG [11]. Radiographic evaluation often reveals small flecks of calcification which aids in diagnosing the peripheral ossifying fibroma [11].

Hemangiomas are congenital lesions, and some vascular malformations increase in size during childhood. Characteristic features are brisk bleeding, increased warmth of the tissue, and blanching upon palpation [12]. These findings were not appreciable in our case.

There are two histological variants for the PG. The first one is characterised by the proliferating blood vessels organised in lobular aggregates called LCH PG whereas the second type reveals highly vascular proliferation that resembles granulation tissue called non-LCH PG [9].

The treatment of the PG involves the removal of irritating factors and surgical excision of the lesion in order to reduce recurrences. In the present case, the patient was subjected to an excisional biopsy of the lesion. Early detection of the PG results in a more conservative treatment and management. The other treatment options for the management of intraoral PG include laser-assisted excision [13] and intralesional injections of corticosteroids [14]. However, surgical excision is the most preferred and commonly used method and regular follow-up is recommended as there is a high chance of recurrence of oral PGs [13].

## Conclusions

Careful medical history followed by a complete clinical, radiological, and histopathological examination is a critical step in the diagnostic process which helps in correct diagnosis and an appropriate treatment plan. In our case, the child was referred to multiple places without treatment as the physicians were unable to correctly identify and treat the lesion as it presented with similarities to PGCG as well. Intraoral PG, although rare, can present in young children as well and if treated promptly reduces the possibility of recurrence and incidence of morbidity. 
